# Translational Pitfalls in SCI Bladder Research: The Hidden Role of Urinary Drainage Techniques in the Rat Model

**DOI:** 10.3390/biology14080928

**Published:** 2025-07-23

**Authors:** Sophina Bauer, Michael Kleindorfer, Karin Roider, Evelyn Beyerer, Martha Georgina Brandtner, Peter Törzsök, Lukas Lusuardi, Ludwig Aigner, Elena Esra Keller

**Affiliations:** 1 Department of Urology and Andrology, Landeskrankenhaus—University Clinic, Paracelsus Medical University, 5020 Salzburg, Austria; 2 Institute of Molecular Regenerative Medicine, Paracelsus Medical University, 5020 Salzburg, Austria; 3 Department of Pediatric and Adolescent Surgery, Landeskrankenhaus—University Clinic, Paracelsus Medical University, 5020 Salzburg, Austria; 4 Faculty of Health and Sport Sciences, Széchenyi István University, 9026 Győr, Hungary

**Keywords:** neurogenic lower urinary tract dysfunction, spinal cord injury, translation, rat model, detrusor hypertrophy, bladder wall thickness, vesicostomy, wound management

## Abstract

Spinal cord injury (SCI) frequently leads to neurogenic lower urinary tract dysfunction, for which appropriate bladder management is essential. While clinical care relies on continuous low-pressure drainage in the acute phase, rat models commonly use twice-daily manual bladder expression—a method known to generate high intravesical pressures and retention. This study evaluated the impact of this standard practice on bladder tissue remodeling by comparing it to continuous drainage via high vesicostomy in a rat SCI model. 32 female Lewis rats underwent thoracic contusion SCI and were assigned to either manual expression or vesicostomy-based bladder management. Over eight weeks, locomotor recovery, wound healing, and bladder histology were assessed. Vesicostomy proved technically simple but required tailored wound care and calibration. Results showed significantly greater bladder wall thickness, detrusor muscle hypertrophy, urothelial thickening, collagen deposition, and mast cell infiltration in the manual expression group compared to both vesicostomy and controls. In contrast, vesicostomy animals exhibited near-control levels across most parameters. These findings highlight that commonly used bladder emptying protocols in rat SCI models may overestimate structural bladder changes and inflammatory responses. Refined drainage strategies such as vesicostomy can minimize secondary damage and improve the translational relevance of preclinical SCI research.

## Simple Summary

Spinal cord injury (SCI) often leads to problems with bladder control, making proper bladder care very important. In hospitals, patients usually receive constant bladder drainage through a catheter to avoid pressure and damage. In animal research, however, bladder emptying is often performed by hand twice a day. This method can cause the bladder to stretch too much and build up pressure, which may lead to changes in the bladder tissue that are not caused by the injury itself. In our study, we compared two methods of bladder care in rats with SCI: the common hand-emptying technique and a surgical method called vesicostomy, which allows urine to drain freely. We looked at how each method affected the bladder over eight weeks. We found that the hand-emptying method caused much more thickening and scarring of the bladder, as well as signs of inflammation. Despite the same type of injury, the vesicostomy group showed much less damage, almost like healthy rats. Our results show that the way bladder care is handled in animal studies can strongly influence outcomes. More natural drainage methods like vesicostomy may give more accurate results and help future research better reflect real-life conditions in patients.

## 1. Introduction

More than 80% of individuals experience neurogenic lower urinary tract dysfunction (NLUTD) following a spinal cord injury (SCI) [1]. Spinal cord lesions located above the sacral micturition center are typically associated with detrusor overactivity, elevated intravesical pressures, and detrusor-sphincter dyssynergia. In contrast, lesions at or below the sacral level generally lead to a hypo- or acontractile detrusor. Effective and individualized bladder management from the early stages of care is critical for optimizing long-term outcomes [2]. During the phase of acute spinal shock, when the bladder is typically acontractile, management strategies include the use of an indwelling catheter or regular clean intermittent catheterization (CIC) [1,3].

Until the 1970s, urological complications were the leading cause of death after SCI [4]. Over the subsequent decades, urology-related mortality was reduced by half. Today, urological causes rank fourth among the causes of death in individuals with SCI [4,5]. A major factor contributing to this decline was the shift in bladder management to clean intermittent catheterization, which proved to be the safest method with the lowest potential for urological complications [6,7,8].

Current neuro-urological research is focused on identifying therapeutic strategies to prevent the development of NLUTD. The rat is the predominantly used animal model in neuro-urological SCI research, as it provides a reliable model for SCI lesions and allows feasible urodynamic assessments and functional evaluations [9,10,11,12].

However, bladder research in the rat model faces certain limitations. Especially in the primary phase after the SCI, the management of human and rat bladder diverge immensely. In clinical practice, human patients receive a transurethral indwelling catheter immediately after SCI to ensure bladder drainage [13,14]. In contrast, continuous or intermittent catheterization is not feasible in rats. Instead, the standard method for bladder emptying during the spinal shock phase or until spontaneous voiding returns is manual expression using the Credé maneuver twice daily. This approach is not physiologically appropriate, considering that rats typically void every 8–10 min [15]. Independently from the cause, urinary retention promotes bladder over distension and secondary damage to the bladder [16,17]. High-pressure chronic retention itself causes detrusor hypertrophy and connective tissue infiltration [18,19,20]. As in humans, rats lose their natural micturition reflexes after an SCI due to the spinal shock. They go into retention and overflow incontinence due to a lack of assisted bladder emptying and receive a high-pressure manual expression of their bladder twice a day. This may influence the frequently reported marked increase in bladder filling volume, as recently described by Ferreira et al., from 0.5 mL in healthy animals up to 9 mL seven days after SCI [21].

These conditions alone may induce functional and histological changes in the bladder wall, which raises the question whether the rat bladder management after SCI itself alters the bladder wall, separate from the SCI related changes. This on the other hand could indicate a strong bias for the testing of new therapy approaches in the rat model so far.

While neuro-urological care following SCI has been significantly optimized in humans, the rat model still relies on the outdated and proven suboptimal Credé method for bladder management [6], forming the basis for current experimental therapeutic investigations.

Our study aims to test the hypothesis that a pressure-free bladder management approach, designed to prevent retention and avoiding manual expression, leads to different urinary bladder outcomes after suprasacral SCI in rats compared to the standard method. For this purpose, a vesicostomy was used as an appropriate urinary diversion immediately after SCI surgery. Following a contusion injury in rats, we compared standard bladder management (manual expression) to the high vesicostomy, as method that permits spontaneous bladder filling and drainage via an overflow mechanism. Main focus was put on the SCI–bladder-related structure with or without retention and manual expression.

## 2. Materials and Methods

This study was performed under the protocols approved by the Austrian Animal Testing Commission of the Federal Government (BMBWF, according to the Directive 2010/63/EU, Section 35 TVG 2012) with the research numbers BMWFW-2022-0.062.928 and BMWFW-2023-0.848.340. We certify that all applicable institutional and governmental regulations concerning the ethical use of animals were followed.

### 2.1. Animals

In total, 32 female, 3-month-old strain Lewis rats (Charles River Laboratories, Sulzfeld, Germany) were used for this study.

Upon arrival, all animals underwent two weeks of acclimatization and handling. In an initial pilot trial, 12 randomly assigned animals were included to assess issues of vesicostomy regarding wound management and feasibility. Six healthy animals underwent a vesicostomy (VES) and six animals underwent a spinal cord contusion injury at thoracic segmental level T9 and a vesicostomy (SCI + VES). Animals of the pilot study were followed up for 56 days.

In the consecutive study, a total of 20 rats were subjected to one of the following three groups: healthy control without surgical intervention (CT; *n* = 6), spinal cord contusion injury with standard bladder management (SCI; *n* = 7), and spinal cord contusion injury with vesicostomy (SCI + VES; *n* = 7). The character of the study was purely explorative without blinding and no integrated sham-SCI groups. Animals of the experimental study were followed up for 56 days. One rat of each group had to be excluded (CT group (*n* = 1): loss of tissue during paraffin embedding; SCI (*n* = 1): mismatch of SCI severity; SCI + VES: died during first days post injury (dpi) (autopsy did not reveal any clear cause).

From the end of the acclimatization phase until the end of the follow-up period, rats were placed in a playpen unit for their comfort and quality of life once weekly. Only during the first week after surgery was no playpen scheduled.

### 2.2. Vesicostomy

**Figure 1:**
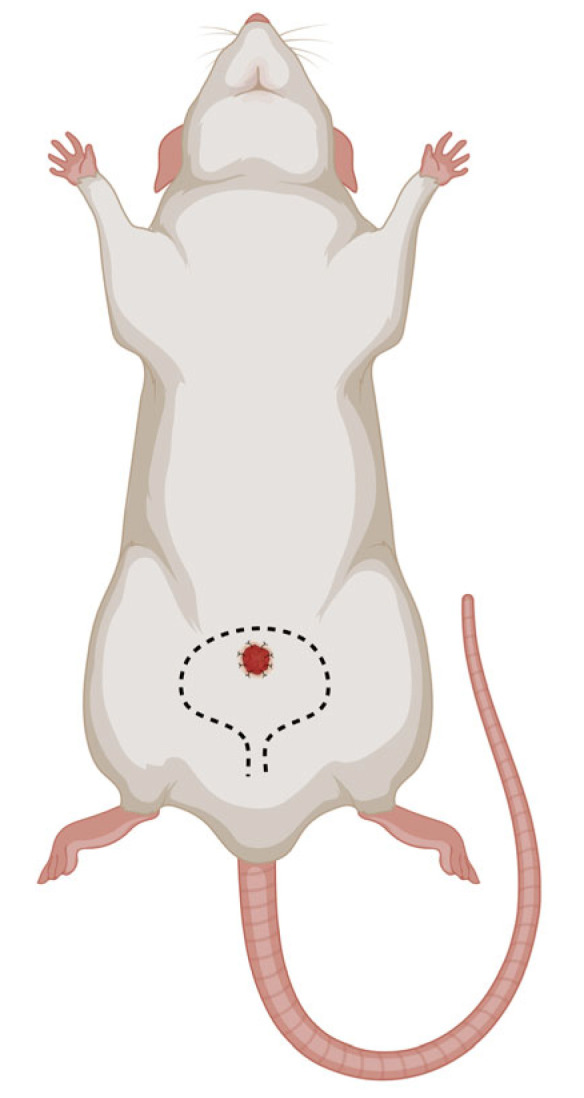
Image showing a rat in supine position with the vesicostomy in place (created with www.biorender.com (accessed on 4 June 2025)).

In general anesthesia (mixture of medetomidine (0.15 mg/kg), midazolam (0.08 mg/kg) and fentanyl (0.01 mg/kg), intramuscularly), the rat was placed in supine position. Clean generous shaving around the operation field helped for later wound healing. The skin was opened with a small 3 mm midline incision at the height of the bladder (clinically knee-high midline incision). Blunt preparation of the abdominal muscle layers and careful incision of the peritoneum opened the abdominal cavity. The naturally filled bladder was seized by a small surgical forceps and carefully pulled through the skin incision. This allowed a precise assessment of the bladder and identification of the bladder dome. The vesicostomy was positioned on the ventral bladder wall about 2–3 mm under the bladder dome. Two holding sutures with 5–0 Vicryl suture in the bladder wall were set to the left and right of the planned bladder incision to keep the bladder in position during the incision. The length of the bladder incision was one millimeter. Inside-out single knot sutures (5–0- Vicryl suture) tied the opened bladder to the abdominal wall at 2′, 4′, 6′, 8′, 10′ and 12′ o’clock. If the skin incision and the vesicostomy matched, no further skin suture was needed and the vesicostomy was situated at the level of the abdominal wall (Figure 1).

After the procedure, the skin was washed with a mucosa suitable agent (ActiMaris^®^ FORTE Wound Irrigation Solution, Appenzell, Switzerland). As a last step, a skin adhesive glue (Dermabond Advanced, Ethicon, Johnson & Johnson Medical GmbH, Norderstedt, Germany) was placed around the vesicostomy to enable wound healing despite urine around the fresh wound. To keep the glue from the bladder and note the vesicostomy size, a calibration stab was placed in the vesicostomy during the last step. The procedure took about 15 min. Post-surgical analgesics and antibiotics included Meloxicam (2 mg/kg, daily, s.c.) and enrofloxacin (1.5 mg/kg, daily, s.c.), which were administered during the first five days post-surgery together with Nutri-Cal (500 µL, daily, oral) as a food supplement and 0.1 molar sucrose in drinking water.

### 2.3. Spinal Cord Contusion Injury

Within the same general anesthesia (mixture as above), a standard laminectomy was performed at thoracic segmental level T7-T10. To create a severe contusion at the thoracic segmental level T9, the spinal cord was impacted with 250 kilodyne of force by an Infinite Horizon (IH)-0400 Impactor (Precision Systems and Instrumentation, Fairfax Station, VA, USA).

Intactness of the dura mater then was controlled and thereafter the skeletal muscle layers were sutured up in layers and the skin was closed. Post-surgical analgesics and antibiotics were administered as described above. In the group without vesicostomy, the bladders were emptied manually twice daily in a standard manner. Urine volumes, discoloration and clarity were noted in a bladder diary for all groups.

### 2.4. Wound Management and Vesicostomy Management

In the first week after the procedure, the team controlled the wounds twice daily and checked the animals for signs of pain and discomfort according to the Grimace Scale [22]. Calibration of the vesicostomy was performed every two days. When the skin adhesive glue started to break away between 5 and 7 dpi, the team removed the remains together with the skin clamps after SCI in a short ventilation anesthesia (1% isoflurane in O2 flow). Daily skin washing with the mucosa suitable agent (ActiMaris^®^ FORTE Wound Irrigation Solution, Appenzell, Switzerland) prevented further infection. To form a suitable barrier between the skin and the dripping urine, a skin protectant agent (3M™ Cavilon™ Advanced Skin Protectant, 3M, Neuss, Germany) was applied to the abdominal skin on demand. After stabilization of the wound situation, we performed daily washing and moisturized the skin with a mainly oil-based care (Filme olio, HULKA S.r.l., Rovigo, Italy) to support natural skin rehabilitation with additional barrier function as a standard of care.

The wound management was designed with an expert for wound healing and incontinence-associated dermatitis. All animals stayed under antibiotic low dose therapy (0.50 mg/kg, daily, s.c.) during the follow up, starting at day 21. If signs of infection around the wound arose, antibiotic dosing was increased to standard dose (1.5 mg/kg, daily, s.c.) for 5 days.

### 2.5. Calibration of Vesicostomy

Calibration of the vesicostomy was performed every day in the awake rat. Due to the lack of mid-abdominal sensation post SCI, this was easily feasible in the awake animal. Calibration stabs (Charriere sizes 6–10) were inserted starting with sizes individually fitted to the individual’s vesicostomy. From 29 DPI on, calibration was performed every two days.

### 2.6. BBB Locomotor Score

For testing their ability to use the hind limbs after SCI, two observers scored the free moving rats according to the 21-point BBB locomotion scale [23]. The analysis included paw placement, weight support, coordination of limbs and stepping, and joint movement, as well as trunk stability and tail position. The tests were performed at baseline and 1 day, 4 and 8 weeks after SCI.

### 2.7. Perfusion and Histology

At day 56 of the follow up, animals were deeply anesthetized (mixture of Ketamine (273 mg/kg), Xylazine (7.1 mg/kg) and Acepromazine (0.625 mg/kg)) and transcardially flushed with ice cold heparinized saline (0.9% NaCl with 10 units/mL Heparin) until no blood discard was observable. The bladder was removed before the animal underwent subsequent perfusion fixation with ice cold 4% paraformaldehyde (PFA) dissolved in 0.1 M PO4 buffer.

The bladder was weighted, then opened from the neck to the dome and divided into halves for further analysis. One half of the bladder wall (1 × 1 cm) was fixed in a cell capsula (CellSafe + Biospy capsula, CellPath, Newton, UK) to ensure a straight fixation for bladder wall diameter analysis. To allow reliable and comparable measurements, all tissues were harvested natively and embedded using a standardized protocol that involved positioning the opened bladder in a sponge bed to prevent compression, tension, or curling of the tissue. The other half of the bladder wall was stored in RNAlater and stored at −80 °C.

Bladders were post-fixed in 4% PFA for 1 h before being thoroughly washed and stored in phosphate-buffered saline (PBS) with 0.01% sodium azide at 4 °C until further processing.

Bladder specimens were embedded in paraffin via an increasing ethanol series (70% to 100%), methylbenzoat and butylacetat (all chemicals Carl Roth, Karlsruhe, Germany), and serially sectioned at 10 µm. For histological examinations of the smooth muscle, connective and urothelial tissue, Masson-Goldner trichrome staining was utilized. The stained sections were photographically documented on a Slide Scanner (Olympus VS120, Olympus Europa SE & Co. KG, Hamburg, Germany), followed by a semi-quantitative morphometrical analysis of the smooth muscle, connective and urothelial tissue content using Fiji (ImageJ version 1.54f, Bethesda, MD, USA). Bladder wall thickness was measured at three positions along the bladder section where the whole bladder diameter was clearly visible. Markers were set using Fiji analysis tools. For collagen distribution and quantity, Picro Sirius Red staining was performed, followed by quantitative measurement of the collagen signal via Fiji analysis threshold settings. Mast cells were stained with a mast cell tryptase kit (Combined Eosinophil-Mast Cell stain kit, ab 150665, Abcam, Cambridge, United Kingdom). Mast cells were photographically documented on the Slide Scanner (Olympus VS120, Olympus Europa SE & Co. KG, Hamburg, Germany) and counted by two independent persons.

### 2.8. Statistics

Experimental data were processed by a spreadsheet and statistics tool (GraphPad Prism 10.2.0; GraphPad Software, La Jolla, CA, USA, www.graphpad.com (accessed on 4 June 2025) to compute mean values, standard deviations, and statistical significance. Pairwise comparison was performed by unpaired *t*-test. A comparison of multiple datasets was achieved by an ordinary One-way ANOVA. *p* values ≤ 0.05, ≤0.01, ≤0.001 and ≤0.0001 were considered significant and marked in the artwork accordingly (*, **, *** and ****).

## 3. Results

This animal trial was designed as a two-phase study. An initial set of animals was used to define standard operating procedures of the vesicostomy, calibration and wound management. The second part focused on bladder-related structural changes in animals with either standard bladder management or vesicostomy after SCI.

### 3.1. Spinal Cord Injury and Wound Management After Vesicostomy

#### 3.1.1. No Difference in Groups According to the Contusion and Locomotor Scores

**Figure 2:**
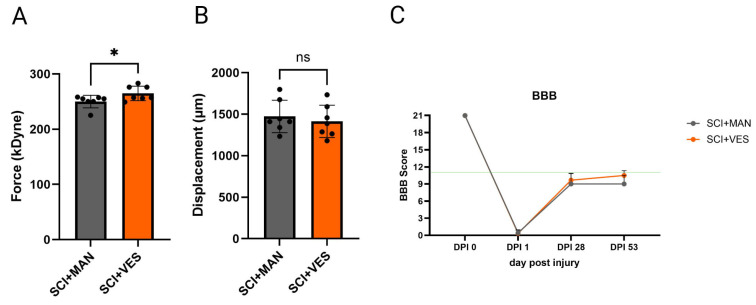
Infinite Horizon Impactor parameters for spinal contusion. (**A**) Actual force (in kilo Dyne); (**B**) displacement of the spinal tissue (in micrometer); (**C**) locomotor score (BBB scale) over 53 days post injury (dpi). ns: not significant and *: *p* ≤ 0.05. Groups: Spinal cord injury with manual expression (SCI + MAN); spinal cord injury with vesicostomy (SCI + VES). Green line: below no coordinated walking.

Directly after the impact with the contusion rod of the IH impactor, the actual force and tissue displacement were noted and later correlated between the groups. The actual force was significantly different between the two groups (pairwise comparison, unpaired *t*-test, two-tailed, *p* = 0.043, Figure 2A) with more actual force seen in the SCI group with vesicostomy. The tissue displacement analysis showed no significant difference (pairwise comparison, unpaired *t*-test, two-tailed, *p* = 0.581, Figure 2B). The locomotor score, measured before, 1 day, 4 and 8 weeks after SCI, showed intact locomotor behavior pre-injury, a clear drop to no or slight joint movement one day after SCI, and only minor recovery over 2 months post SCI with no regaining of coordinated stepping patterns in both SCI groups. At two months post SCI, the difference in the locomotor score (BBB score) was not significant (pairwise comparison, unpaired *t*-test, two-tailed, *p* = 0.254, Figure 2C).

#### 3.1.2. Wound Management Is Key for Good Healing and Bladder Drainage

Immediately after the procedure, wound management was initiated to ensure optimal healing and bladder drainage. The skin around the newly created vesicostomy (see Figure 1) was cleaned with a mucosa-compatible agent (ActiMaris^®^ FORTE Wound Irrigation Solution, Appenzell, Switzerland). A skin adhesive (Dermabond Advanced, Ethicon, Johnson & Johnson Medical GmbH, Norderstedt, Germany) was then applied around the vesicostomy to shield the wound from urine exposure and promote healing over the subsequent 5–7 days. To prevent adhesive migration into the bladder and to maintain vesicostomy diameter, a calibration stab was inserted during this final step.

In the first post-operative week, wound monitoring was conducted twice daily, with regular assessments for pain or discomfort. Vesicostomy calibration was repeated every two days. As the adhesive began to detach between days 5–7 post-injury, residual glue and skin clamps were removed under brief anesthesia. Daily cleaning with ActiMaris Forte continued to prevent secondary infections.

To protect the abdominal skin from urine exposure, a barrier film (Cavilon Advanced, 3M) was applied as needed. Once wounds had stabilized, the protocol transitioned to routine cleaning and moisturization using oil-based products (Filme olio, HULKA S.r.l., Rovigo, Italy) to promote epithelial recovery and strengthen the skin barrier.

The protocol was developed in collaboration with a wound healing specialist, with all animals receiving low-dose antibiotic prophylaxis throughout the follow up period. If infection was suspected, antibiotics were upscaled for five days. This approach proved critical to maintaining vesicostomy function and ensuring consistent bladder drainage without complications.

### 3.2. Bladder Histology

#### 3.2.1. Spinal Cord Injury Impacts Bladder Thickness, but Clearly Less When Drained via a Vesicostomy

**Figure 3:**
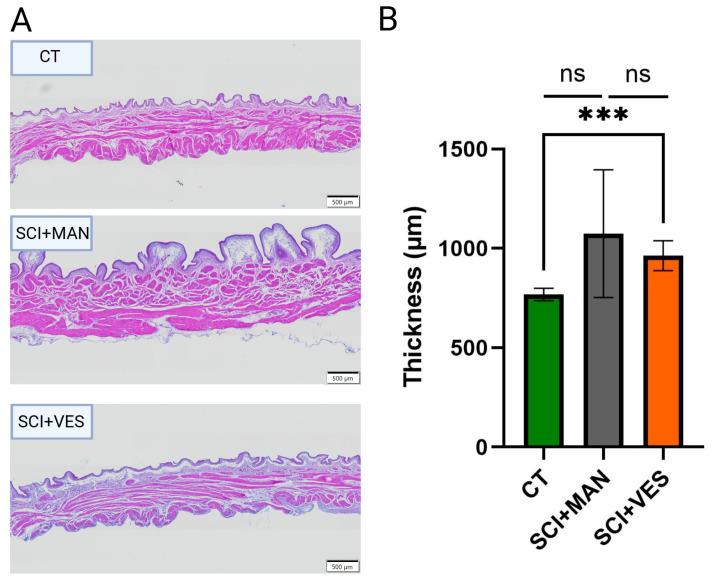
(**A**) Bladder wall images of one representative rat per group: control (CT), spinal cord injury groups with manual expression (SCI + MAN) and spinal cord injury with vesicostomy (SCI + VES). (**B**) Bladder wall thickness (in micrometer) per group. ns: not significant and *** *p* ≤0.001.

Bladder wall thickening is one of the most obvious changes post SCI. Bladder wall thickness increased after SCI, with a higher mean increase in the SCI groups with manual bladder expression than the SCI group with vesicostomy (Figure 3A). The difference amongst all three groups was not significant (One-way ANOVA, *p* = 0.071, Figure 3B). The direct comparison of the SCI group with manual expression to the control group (pairwise comparison, unpaired *t*-test, two-tailed, *p* = 0.0624) as well as the comparison of the two SCI groups revealed no significant difference (pairwise comparison, unpaired *t*-test, two-tailed, *p* = 0.4255) and a significant difference between the SCI group with vesicostomy and the control group (pairwise comparison, unpaired *t*-test, two-tailed, *p* = 0.0035).

#### 3.2.2. Muscle and Uroepithelial Tissue Are Mostly Impacted by High Intravesical Bladder Pressures and Manual Expression Technique

**Figure 4:**
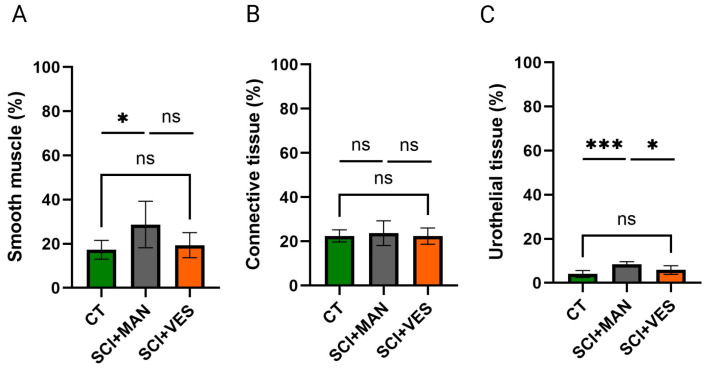
Analysis of smooth muscle (**A**), connective (**B**) and urothelial (**C**) tissue in percentage for the three distinct groups. ns: not significant; *: *p* ≤ 0.05 and *** *p* ≤ 0.001. Groups: spinal cord injury with manual expression (SCI + MAN); spinal cord injury with vesicostomy (SCI + VES); Control group (CT).

The three main tissue types of the bladder wall were analyzed by a semi-quantitative morphometric analysis to correlate the percentages between groups. There was a significant difference in smooth muscle percentage amongst the three groups (One-way ANOVA, *p* = 0.044, Figure 4A) with the highest percentage in the SCI group with manual expression. Pairwise comparisons highlighted a significant increase between the control and SCI group with manual expression (unpaired *t*-test, two-tailed, *p* = 0.047), while the comparison between the control and SCI group with vesicostomy was not significant (unpaired *t*-test, two-tailed, *p* = 0.51). The two SCI groups are discernibly different, with the vesicostomy group being closer to the control group (unpaired *t*-test, two-tailed, *p* = 0.080). Comparisons of the connective tissue showed no significant differences amongst the three groups (One-way ANOVA, *p* = 0.83, Figure 4B), nor for any of the three pairwise comparisons (unpaired *t*-test, two-tailed, control group vs. SCI, *p* = 0.66; control group vs. SCI group with vesicostomy, *p* = 0.97; SCI vs. SCI plus vesicostomy, *p* = 0.63). The uroepithelial tissue percentage was highly different between the three groups (One-way ANOVA, *p* = 0.0007, Figure 4C) with the highest percentage in the SCI group with manual expression. Pairwise comparisons highlighted a significant difference in the SCI group with manual expression to the control group (unpaired *t*-test, two-tailed, *p* = 0.0002) and between the two SCI groups (unpaired *t*-test, two-tailed, *p* = 0.011). No significant difference was observed between the SCI group with vesicostomy and the control group (unpaired *t*-test, two-tailed, *p* = 0.13).

#### 3.2.3. Significantly Lower Collagen Levels Detected Post Spinal Cord Injury with Vesicostomy

**Figure 5:**
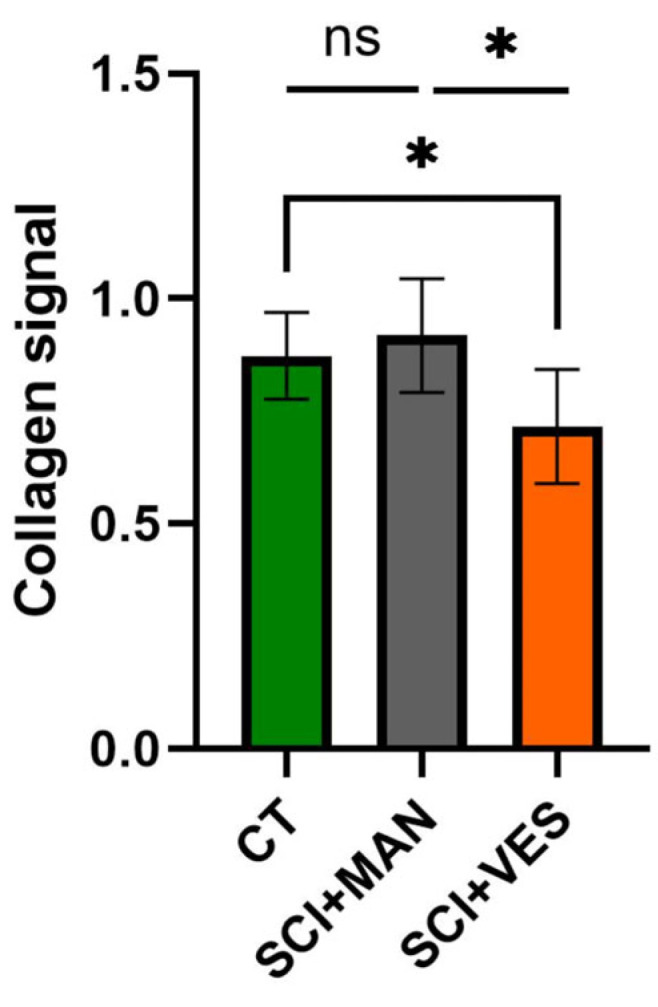
Collagen signal in total bladder wall tissue for all three groups. ns: not significant and *: *p* ≤ 0.05. Groups: spinal cord injury with manual expression (SCI + MAN); spinal cord injury with vesicostomy (SCI + VES); Control group (CT).

Collagens were chosen as representative group to describe the mechanical properties of the bladder. There was a significant difference amongst all three groups (One-way ANOVA, *p* = 0.020, Figure 5) with more collagen in the SCI group with manual expression and less collagen in the SCI group with vesicostomy compared to the control group. Pairwise comparisons showed no significant difference between the SCI group with manual expression and the control group (unpaired *t*-test, two-tailed, *p* = 0.49), while the collagen percentage in the SCI group with vesicostomy was significantly reduced compared to the control group (unpaired *t*-test, two-tailed, *p* = 0.016). Furthermore, the two SCI groups differed significantly (unpaired *t*-test, two-tailed, *p* = 0.037).

#### 3.2.4. Continuous Bladder Drainage Through a Vesicostomy Reduces the Invasion of Mast Cells into the Bladder Tissue

**Figure 6:**
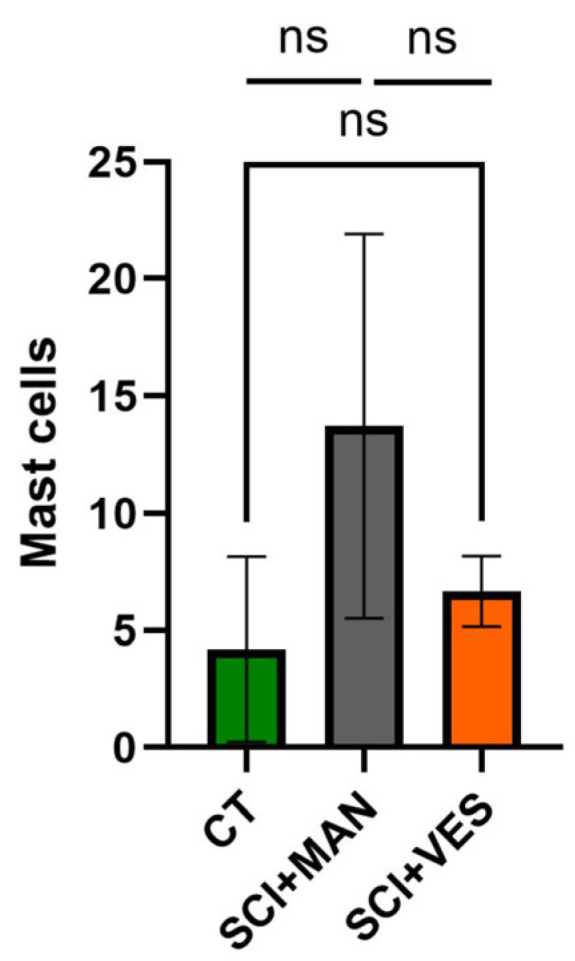
Number of mast cells in bladder wall tissue in all three groups. ns: not significant. Groups: spinal cord injury with manual expression (SCI + MAN); spinal cord injury with vesicostomy (SCI + VES); Control group (CT).

Mast cells were used as an indicator for bladder tissue inflammation. There was a significant difference amongst all three groups (One-way ANOVA, *p* = 0.025) with the highest number of mast cells in the SCI group with manual expression (Figure 6). A pairwise comparison between groups highlighted the highest number of mast cells in the SCI group with manual expression (unpaired *t*-test, two-tailed, *p* = 0.039) and only a marginal increase in the SCI group with vesicostomy (unpaired *t*-test, two-tailed, *p* = 0.19). The difference between the two SCI groups showed a clear trend for higher mast cell number with manual expression (unpaired *t*-test, two-tailed, *p* = 0.064).

## 4. Discussion

The primary objective of this preclinical study was to determine whether the prevention of bladder retention following SCI exerts a significant impact on bladder tissue integrity in the rat model. Specifically, the study aimed to evaluate whether continuous bladder drainage via vesicostomy provides a meaningful improvement over the currently practiced method of twice-daily manual bladder expression.

The animals used in our study formed homogeneous cohorts with consistent spinal lesions and similar degrees of locomotor impairment. Vesicostomy proved to be a rapid and straightforward procedure, performable within 15 min after SCI surgery, requiring minimal technical expertise. However, post-operative wound care demanded specific measures to ensure favorable outcomes. Direct skin protection during the first post-operative week was essential, and vesicostomy patency required initial daily, later bi-daily calibration. Once healing was complete, vesicostomy provided a stable, low-maintenance method of bladder drainage without major complications, and was successfully integrated into the experimental protocol.

Only female rats were used in this study, as the longer urethra in males complicates manual expression after SCI, potentially leading to insufficient bladder emptying. With urine residuals remaining in the bladder, there is a higher risk of subsequent urinary tract infections and exclusion from the trial [24]. Vesicostomy, however, might overcome this issue and represent as a drainage method equally applicable for both sexes, therefore enabling studies in male rats as in female rats. This is particularly valuable, as a general difference in therapeutic success can be expected between the sexes [25]. Currently, preclinical studies are mainly conducted using female rats, despite the fact that the majority of human SCI patients are male.

Moreover, the primary advantage of vesicostomy—continuous drainage—also addresses key limitations of rodent SCI models. While larger animals allow transurethral catheterization consistent with clinical practice [1,13,14,26], rat bladders are typically managed with twice-daily manual expression [12,15]. This contrasts sharply with the rat’s physiological micturition rhythm during the active (dark) phase with voiding intervals of 3–8 min [15,27]. To approximate physiological conditions, 18 to 38 manual expressions would be required per active period [27]—a practically unfeasible approach.

This mismatch likely leads to bladder overdistension and high intravesical pressure post-SCI. It highlights a translational gap in current rodent models: the inability to implement continuous, physiological bladder drainage. As bladder management is typically only mentioned methodologically—and described in detail only in rare cases (e.g., Kleindorfer et al. [12])—it remains difficult to adequately discuss and compare findings across the literature. Manual expression mimics the obsolete Credé maneuver, which is no longer clinically used due to associated risks such as upper tract damage and renal impairment [6].

Our findings clearly demonstrate that modifying post-operative bladder management—specifically avoiding retention and manual expression—led to markedly different outcomes after SCI.

Our primary endpoints were bladder wall thickness and detrusor muscle hypertrophy, parameters highly susceptible to retention-induced changes. The literature consistently reports these alterations post-SCI [28,29,30], but direct comparison is limited by variation in embedding protocols and insufficient methodological detail. We employed a sponge bed within a cell capsule for fixation, yielding high intra-study consistency. Our findings confirm a general increase in bladder wall thickness after SCI; however, it is more prominent for low-frequency manual expression compared to high-output drainage via vesicostomy. This is likely due to both prolonged filling and high pressures during expression. Thus, bladder wall thickening may be less severe in the absence of these factors than previously assumed in the literature.

Semiquantitative morphometric analysis revealed that detrusor hypertrophy occurs even under low-pressure conditions, but to a significantly reduced extent compared to standard manual expression. Given its frequent use as a key outcome parameter in preclinical trials [21,28,31,32], future studies should prioritize physiological drainage methods that avoid retention and pressure spikes.

We also observed urothelial thickening, particularly in the manual group. As comparable findings are scarce in the literature, it remains unclear whether these changes stem from SCI, drainage method, or both. Some studies report early umbrella cell disruption, decreased transepithelial resistance, and increased permeability after SCI [33,34,35], but none describe quantitative urothelial thickening. Future studies should address this gap through systematic histological and molecular assessments of urothelial structure and function [33,35,36].

In terms of connective tissue, total collagen content varied between groups—elevated in the manual group and reduced in the vesicostomy group. Our conclusions are limited to Picrosirius Red staining, which reflects total collagen but not its subtypes. While Herovici staining may provide this differentiation [26], it is not reliable in rats. Hence, robust fibrosis assessment requires a multimodal histological and molecular approach.

To evaluate chronic inflammation, mast cells were quantified. Counts were highest in the manual expression group, followed by vesicostomy and controls. These findings suggest that following successful wound closure, continuous drainage attenuates chronic inflammation post-SCI and that vesicostomy itself does not contribute to it.

Despite these encouraging results, one of the main limitations preventing widespread adoption of the vesicostomy approach is the initial time investment and the complexity of wound management, particularly during the early post-operative phase when complications can occur.

## 5. Conclusions

To date, most SCI bladder research in rats has relied on non-physiological bladder management. Our findings underline the need for feasible, low-pressure, and physiological bladder drainage methods. We advocate for a re-evaluation of standard practices in preclinical models to improve translational reliability and ultimately support novel therapeutic strategies for human patients.

## Data Availability

The data associated with the paper are not publicly available but are available from the corresponding author on reasonable request.

## Abbreviations

The following abbreviations are used in this manuscript:

CIC	Clean intermittent catheterization
CT	Control
dpi	Days post injury
IH	Infinite Horizon
IV	Intravenously
MAN	Manual expression
NLUTD	Neurogenic lower urinary tract dysfunction
PBS	Phosphate-buffered saline
PFA	Paraformaldehyde
SCI	Spinal cord injury
VES	Vesicostomy
